# Synergistic detection of *E. coli* using ultrathin film of functionalized graphene with impedance spectroscopy and machine learning

**DOI:** 10.1038/s41598-025-00121-3

**Published:** 2025-04-30

**Authors:** Amrit Kumar, Shweta Mishra, R. K. Gupta, V. Manjuladevi

**Affiliations:** Department of Physics, Birla Institute of Technology and Science, Pilani (BITS PILANI), Rajasthan 333031 India

**Keywords:** Surface assembly, Surfaces, interfaces and thin films, Biomedical engineering

## Abstract

Bacterial detection and classification are critical challenges in healthcare, environmental monitoring, and food safety, demanding selective and efficient methods. This study presents a novel, label-free approach for E. coli detection using ultrathin Langmuir-Blodgett films of octadecylamine functionalized (ODA)-functionalized graphene on gold electrodes, with a detection range spanning $10^{1}-10^{6}$ colony-forming units/mL (CFU/mL). Electrochemical impedance spectroscopy (EIS) was performed on six bacterial strains, representing Gram-negative and Gram-positive classes, to evaluate selectivity. The method achieved a remarkably low detection limit of 2.5 CFU/mL for E. coli, with its EIS spectra exhibiting distinct features compared to other bacterial strains. The pronounced differences enabled perfect classification using the Bagging Classifier, achieving no false positives. Machine learning (ML) algorithms applied to raw impedance data improved detection precision and reliability, enabling automated and accurate analysis. These findings establish a robust framework for rapid and selective E. coli detection, crucial for ensuring food and water safety. The integration of ML significantly improves detection accuracy, reduces analysis time, and minimizes human error, paving the way for scalable, cost-effective diagnostic tools for diverse biological and environmental applications.

## Introduction

Nanomaterials with tunable size and composition have been engineered for advanced biomedical sensing applications^1^. Among these materials, graphene stands out due to its exceptional electronic and physicochemical properties, which are crucial for the development of various biosensing devices^2,3^. Graphene exhibits unique attributes, including a high specific surface area, excellent biocompatibility, superior electrical conductivity, and remarkable thermal stability, making it an ideal candidate for biosensing platforms^4^. Additionally, its delocalized $\pi$-electrons enable $\pi -\pi$ interactions with bioanalytes, significantly enhancing the sensitivity and performance of these sensors. The high surface area of graphene, coupled with its effective $\pi -\pi$ interactions, facilitates the adsorption of biomolecules, leading to measurable changes in the sensor as these molecules efficiently bind or react on its surface^2^. Furthermore, graphene interacts with a wide variety of organic molecules through non-covalent forces, including hydrogen bonding, electrostatic interactions, van der Waals forces, and hydrophobic interactions. As a result, graphene is considered an excellent material for designing highly specific sensors for diverse analytes, enabling targeted biosensing applications^5–7^.

Dispersing graphene in either an organic solvent or aqueous medium is often essential for film processing and device fabrication. However, pristine graphene exhibits inherent hydrophobicity, which makes its dispersion in solvent challenging. To overcome this, graphene is typically functionalized through a chemical route. Functionalization with various species, including inorganic nanoparticles, organic molecules, and biomolecules, alters the surface chemistry of graphene, enhancing its sensitivity and specificity for applications in biosensing and other fields^6,8–10^. Octadecylamine (ODA) functionalized graphene sheets, where the sheet-to-sheet spacing and stacked morphology are modified by the chain length of the ODA ($\approx$ 2.5 nm), demonstrate enhanced electroactive surface area. Such functionalization not only improves the structural properties of graphene but also increases its sensitivity by enhancing its electroactive surface area^11^.

Several reports have demonstrated enhanced sensing performance by a single layer of graphene compared to bulk graphene films^12,13^. Poonia et al.^12^ observed that ultrathin Langmuir-Blodgett (LB) films of carboxyl-modified graphene (G-COOH) exhibited improved performance for urea detection in aqueous medium compared to thicker spin-coated films. They found that the lowest detectable concentration of urea using LB films of G-COOH (8.3 $\mu$M) was approximately five times lower than that of spin-coated films (41.6 $\mu$M), while the sensitivity of the LB film (42.5 ng/$\text {cm}^2$/$\mu$M) was about three times greater than that of the spin-coated film (12.9 ng/$\text {cm}^2$/$\mu$M). Various techniques exist for fabricating ultrathin films of graphene and its derivatives, including chemical vapor deposition (CVD)^14,15^, dip coating^16,17^, self-assembly^18^, and the LB technique^19–21^. The LB technique stands out due to its superior control over the layer structure and the ability to engineer the surface density, making it particularly advantageous for sensor fabrication. LB technique offers precise control over both the thickness and surface density of the films, which is critical for optimizing sensor performance. In this study, we have employed the LB technique to transfer ODA-graphene (ODA-Gr) layers from the air-water interface onto electrodes for sensing performance measurements. While there are numerous reports on the LB film fabrication of graphene and its derivatives for potential biosensing applications, the biosensing potential of LB film of ODA-Gr has not been extensively explored.

Biosensors for bacterial detection have grown rapidly, with applications in environmental monitoring, food quality, clinical diagnostics, and biological agent identification. Bacterial contamination is a leading cause of hospitalizations and fatalities worldwide, surpassing viruses and hazardous chemicals^22,23^. Despite efforts, deaths from pathogenic bacterial infections are projected to reach 13 million by 2050^24^, and the threat of pathogenic bacteria as biological weapons is concerning^25,26^. Escherichia coli (E. coli), a major foodborne and waterborne pathogen, poses a significant health risk globally, particularly in vulnerable populations^27,28^. Contaminated water sources remain a major issue, with E. coli serving as a key indicator of microbial contamination risk^29^. Thus, the development of rapid and effective detection methods for E. coli is crucial for diagnostic and public health purposes.

Existing E. coli detection methods, such as colony-counting, are labor-intensive, requiring multiple steps, skilled personnel, and extended waiting times of 10-48 hours^30^. The bacterial plating process typically includes a series of biochemical tests (e.g., catalase, citrate, Gram staining, and methylene red staining). While molecular techniques like polymerase chain reaction (PCR) can reduce detection times to hours, they still suffer from limited sensitivity at low bacterial concentrations ($<<$1-100 CFU/mL) and require complex protocols^31^. To enhance specificity and sensitivity, labeled sensors incorporating DNA, antibodies, aptamers, peptides, and phages are employed but remain hindered by time-consuming, costly sample preparation method^32,33^. Optical detection techniques-including surface plasmon resonance, localized surface plasmon resonance, surface-enhanced Raman scattering, and surface-enhanced fluorescence-offer high specificity but face challenges, such as potential peak deflection from strong EM fields and limitations in field deployment due to their size and complexity^34^. Electrochemical Impedance Spectroscopy (EIS) presents a promising alternative as a label-free, highly sensitive, and cost-effective method. EIS can quantify critical interfacial properties-such as diffusion, double-layer capacitance, and analyte-electrode interactions-by modeling impedance data, thus providing a robust platform for bacterial detection. Initial studies have shown the potential of graphene-based EIS sensors for E. coli detection. Jijie et al.^35^ reported EIS detection of E. coli using gold electrodes functionalized with reduced graphene oxide layers and anti-E. coli antibodies, achieving detection limits from $10^{1}$ to $10^{4}$ CFU/mL. Similarly, Gupta et al.^36^ utilized amino-functionalized graphene oxide on electrodes, detecting concentrations from $2\times 10^{2}$ to $10^{8}$ CFU/mL, while Wang et al.^37^. achieved a limit of $1.5\times 10^{2}$ CFU/mL on Au-modified graphene paper electrodes. These findings indicate a need for further investigation into broad-range, label-free EIS methods for sensitive, rapid E. coli detection.

Numerous studies in the literature have reported the detection and differentiation of E. coli using impedance-based measurements. However, our approach in this work highlights the label free sensing mechanism. Most of the impedance based detection for E .coli employs either bio-labels or antigen specific to E. coli, to be fixated on the sensing platform for its selective detection. However, in this article we propose ODA-Gr as the standalone functional layer without any bio-ligand or antigens for specific detection. Here we have employed ultrathin film of ODA-Gr fabricated via LB technique to achieve such a low detection limit and broad detection range which has not been significantly explored as per the best of our knowledge. In this article, we present a novel, label-free, selective sensing approach for E. coli detection using ultrathin LB film of ODA-Gr modified gold electrodes, covering a broad detection range of $10^{1}-10^{6}$ CFU/mL. EIS was performed with six bacterial strains, representing both Gram-negative and Gram-positive classes, to evaluate selective sensing. The technique demonstrated a remarkably low detection limit of 2.5 CFU/mL specifically for E. coli. Typically, EIS sensitivity is analyzed through equivalent circuit modeling, which can be labor-intensive and requires specialized knowledge to accurately interpret analyte-electrode interactions. To streamline this process, we integrated machine learning (ML) algorithms with EIS data analysis. By applying ML classification algorithms to raw impedance magnitude data across frequencies for each bacterial concentration, we enhanced E. coli detection sensitivity and specificity. This ML-based approach reduces the need for circuit fitting, eliminates potential human error, and offers an automated, precise, and robust alternative for selective bacterial sensing^38^.

## Results and discussion

**Fig. 1:**
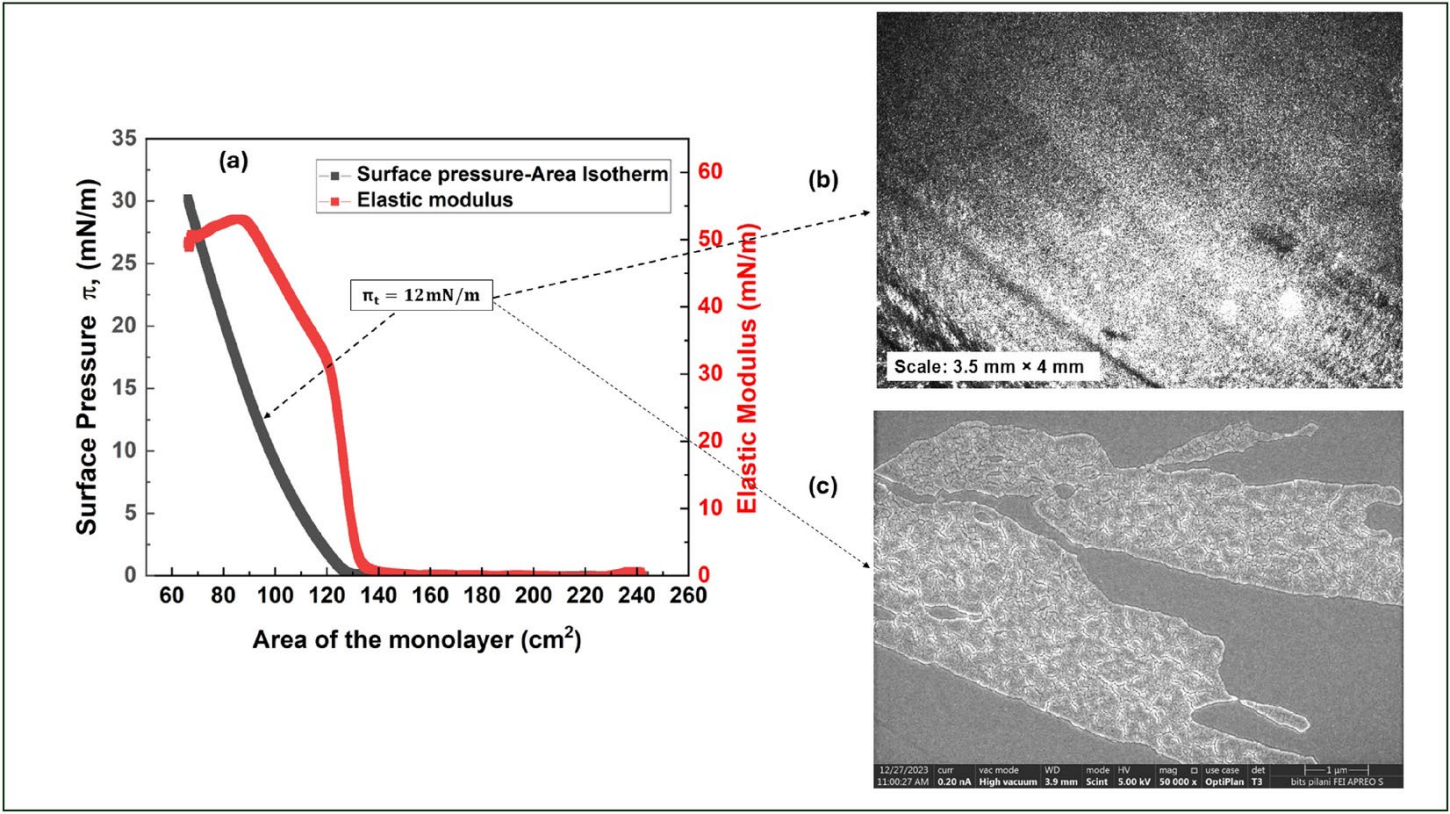
(**a**) Surface pressure ($\pi$) - area isotherm of Langmuir film of ODA-Gr at the air-water interface. Here, *Area* represents the area occupied by the film entrapped between the barriers. The target surface pressure $\pi _{t}= 15mN/m$ is indicated by the arrow. (**b**) The corresponding BAM images at $\pi _{t}$ at the air-water interface (scale = $3.5\times 4mm^{2}$) and (**c**) FESEM image of the ultrathin LB film of ODA-Gr deposited on Si chip at the $\pi _{t}$.

Synthesized powder samples of reduced graphene oxide (rGO) and ODA-Gr are characterized using Xray diffraction (XRD), field emission scanning electron microscope (FESEM) and Fourier transform infrared (FTIR) spectrometer. The ODA functionalization was established from the XRD and FTIR spectra. The characterization results are shown in the Supplementary Information. Further, in order to obtain the LB films on the solid surface of a device, it is essential to investigate the surface phases and behavior of Langmuir film at the air-water interface. The Langmuir film of ODA-Gr was formed at the air-water interface and surface pressure ($\pi$) vs area isotherm was recorded as shown in Fig. 1.

During the compression of the Langmuir film, the surface pressure remains negligible until the surface area reduces to approximately $127~\text {cm}^{2}$ (Fig. 1a). This region of negligible surface pressure typically signifies the coexistence of gas and liquid-like phases within the monolayer, where molecules are sparsely distributed and lack significant interaction. Upon further compression, the surface pressure begins to increase sharply and monotonically as the molecules come closer, indicating a transition toward a more condensed, organized structure. At around $125~\text {cm}^2$, the marked increase in surface pressure signifies the onset of the liquid-like phase in the Langmuir monolayer of ODA-Gr. The film is compressed until it reaches a surface pressure of $30~\text {mN/m}$, creating a densely packed monolayer. The in-plane elastic modulus (E) reaches a peak value of $52~\text {mN/m}$ when the surface pressure is approximately $12~\text {mN/m}$. This surface pressure is selected as the target pressure ($\pi _t$) for the deposition of LB films. A Brewster Angle Microscopy (BAM) image of the Langmuir film of ODA-Gr at the target pressure $\pi _t$ displays a uniform texture (Fig. 1b). The morphology of a single layer of LB film of ODA-Gr was studied using field emission scanning electron microscopy (FESEM) as shown in Fig. 1c. The FESEM image shows a large monolayer flakes of ODA-Gr on the solid substrate.

**Fig. 2:**
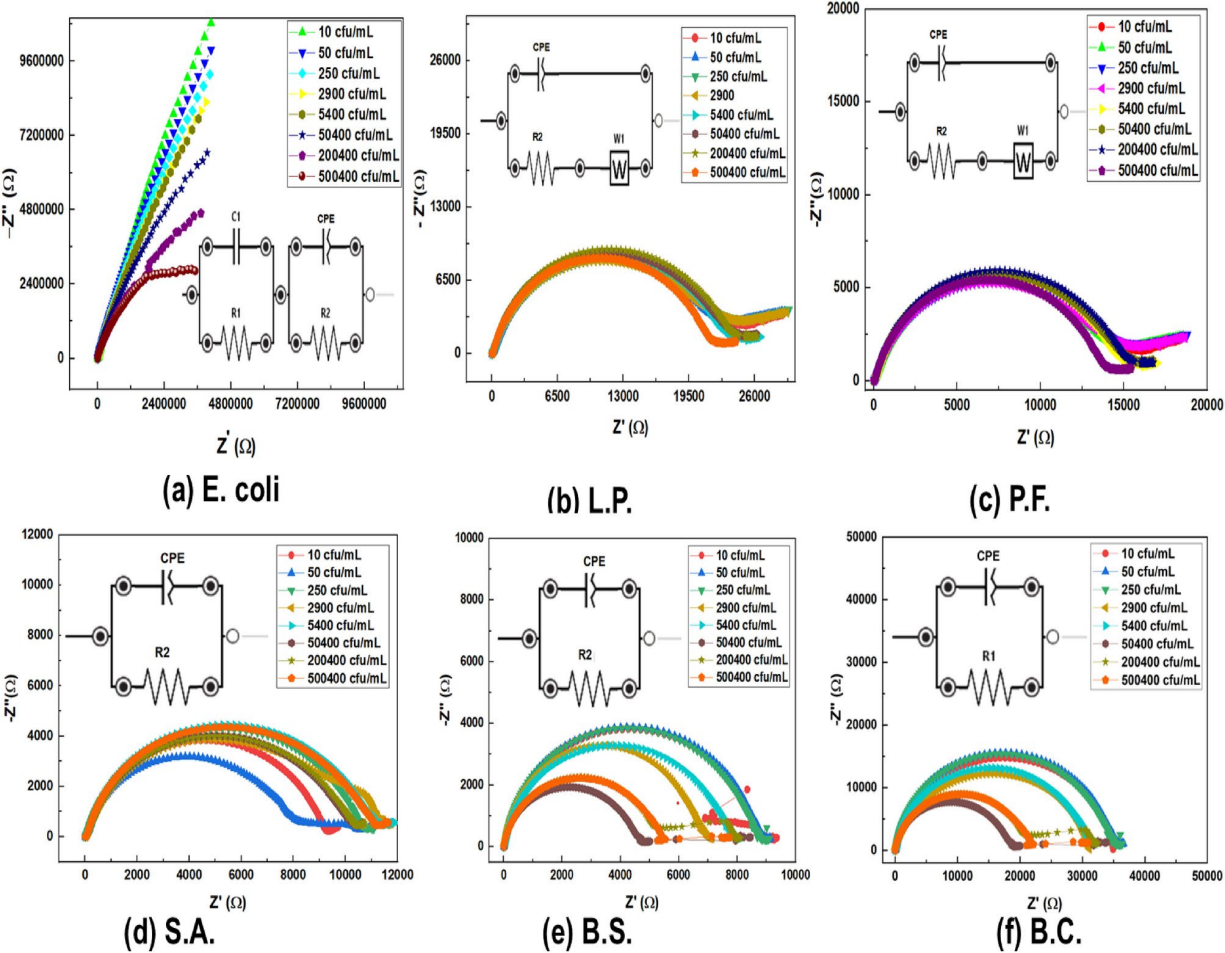
Nyquist plot for wide range of concentration along with the circuit employed for data fitting(inset) for (**a**) E. coli (**b**) L.P. (**c**) P.F. (**d**) S.A. (**e**) B.S. and (**f**) B.C.

The LB film of ODA-Gr was deposited onto the gold electrodes and were utilized for sensing of Gram-positive and Gram-negative bacteria using electrochemical impedance spectroscopy (EIS). The EIS were performed at different concentrations for each of the six bacteria culture. Fig. 2 shows the Nyquist plots for a wide range of concentrations of each bacteria under investigation. The corresponding circuit model employed to fit the observed data is shown in each of the respective plots.

The impedance data showed unique response for E. coli as compared to other class of bacteria. In case of E. coli, arc of semicircle was obtained at the higher frequency regime whereas a complete semicircle was observed in the rest of Gram-negative and Gram-positive bacteria. For the other two Gram-negative bacteria (*e*.*g*. P.F. and L.P.), a small tail is also observed in the lower frequency regime which was absent in the response curve of Gram-positive bacteria. The initial arc towards the high frequency regime (in case of E. coli) indicates capacitive and resistive behavior due to double-layer capacitance and electron transfer resistance at the electrode surface. This section of the impedance curve typically reflects the resistance related to rapid ion transport across the interface, where higher frequencies correspond to faster response times. In other cases of the bacteria, the full semicircles in a Nyquist plot indicates a single, well-defined charge-transfer process in which electrons are transferred across the electrode interface with a uniform double-layer capacitance. The low-frequency tail following the semicircle (for P.E. and L.P.) is typically due to mass transfer limitations, often referred to as Warburg impedance or diffusion impedance. The tail represents the slower diffusion-controlled processes occurring as ions move from the bulk solution to the electrode surface. The tail appears at lower frequencies because diffusion is a slow process compared to charge transfer. The model circuit employed for fitting the data for E. coli, other two Gram-negative bacteria (*i*.*e* P.F and L.P) and the three Gram-positive bacteria are chosen accordingly. From Fig. 2, it is evident that the magnitude of impedance for E. coli is $\sim 10^{3}$ times greater than the impedance magnitude of rest of the bacteria. The impedance response for E. coli with respect to its concentration is also decreasing systematically as compared to other bacteria. Although, E. coli belongs to Gram-negative family, the outer peptidoglycan layer is mono-molecular thick ($\sim 10$) nm when compared to the thickness of other Gram-negative bacteria. In E. coli, the outer and inner membranes adhere to each other at multiple locations referred to as Bayer patches. These patches, numbering several hundred, have the potential to disrupt the continuity of the peptidoglycan layer due to its monolayer thickness. Therefore, when E. coli comes close vicininty to graphene interface due to its interaction, the outer layer may get ruptured. The rupture releases weak organic acids in its extra cellular environment. Such acids alters the local pH in the nano regime gap between the E. coli bacteria and graphene layer leading to increase in the conductance of graphene film. Moreover, conductance of graphene increases monotonically with increasing concentration of E. Coli. This was modelled by including an additional parallel RC circuit consisting of capacitance $C_1$ and resistance $R_1$. Due to the electrostatic interaction between the negatively charged outer membrane of Gram-negative bacteria and the positively charged amine-functional group of the ODA-modified graphene surface, an interfacial double layer capacitance can form. As shown in Fig. 2(a, b, and c), this interaction can be modeled by introducing a constant phase element (CPE) in parallel with charge transfer resistance ($R_2$). Typically, pseudo-capacitance and charge transfer are modeled with a CPE in parallel with a resistance^39^, while charge polarization is represented by Warburg impedance, denoted as *W*. The combination of these two parallel RC circuits, connected in series, effectively describes the interaction between the ODA-Gr film and E. coli.

Further, in the case of P.F. and L.P., although these bacteria also belong to the Gram-negative family, their impedance response differs from that of E. coli. The impedance response indicates the formation of pseudo-capacitance and charge transfer, with a small tail at lower frequencies that suggests polarization of charged species. The presence of pseudo-capacitance and charge transfer indicates electrostatic interactions between the negatively charged cell walls of these Gram-negative bacteria. The low-frequency tail is due to the redistribution of surface charges on the bacterial cells. The cell walls of P.F. and L.P. are comparatively thicker than that of E. coli, and thus, cell wall rupture did not occur under the chosen experimental conditions. At higher voltages or over a wider frequency range, cell wall rupture might also occur in these two bacteria, given that they share similar Gram-negative characteristics with E. coli. In this case, since the cell walls remained intact, surface charge redistribution was observed. Accordingly, the circuit used for fitting their impedance response comprises a constant phase element (CPE) in parallel with a series combination of resistance and Warburg impedance. Here, the CPE represents pseudo-capacitance ($C_{pc}$), the resistor ($R_2$) represents the charge transfer resistance ($R_{ct}$), and the Warburg element (*W*) accounts for the diffusion of charged species in the sensing environment.

For Gram-positive bacteria (S.A., B.C., and B.S.), interactions with the long ODA chains on graphene involve both electrostatic and hydrophobic forces. These bacteria predominantly form large multicellular clusters, leading to distinctive impedance profiles. The unusual transitions in the impedance response are likely due to clustering and biofilm formation, where the correlation between the signal and colony-forming unit (CFU) counts is disrupted due to the loss of unicellularity. Furthermore, Gram-positive bacteria are mostly non-motile, accumulating on the sensor surface and forming islands of microcolonies. These microcolonies supply a negative charge to the ODA-functionalized graphene, moving the system closer to charge neutrality, which is expected to produce a monotonic change in impedance response. However, because the charge transfer from the microcolonies on the graphene surface is spatially non-uniform, a non-linear response is observed. From the Nyquist plot, the circuit employed for fitting suggest only the formation of pseudo-capacitance ($C_{pc}$) and charge transfer resistance ($R_{ct}$) in Gram-positive bacteria. This response contrasts with that of Gram-negative bacteria, where additional circuit elements are required to model the impedance. The Nyquist plot for Gram-positive bacteria also exhibits noise interference in the low-frequency regime (below 10 Hz), likely due to signal instability during measurements. The parameter values viz. $C_{pc}$ and $R_{ct}$ obtained from circuit fitting are shown in Table 1. The Nyquist plot fitting shows an error of less than $2\%$. It can be noted that in case of B.C. the impedance repsonse showed an anamolous effect. The impedance value is greater than the impedance value of the Gram-negative bacteria. As, B.C. produces extracellular polysaccharides and proteins that can form a biofilm and interact strongly with the graphene surface, creating an additional resistive barrier, thus high impedance is observed.^40,41^

**Table 1:** The estimated values of the circuit components used in fitting of Nyquist plot for all six bacteria culture.

Bacteria concentration (C.F.U/mL)	E. coli				P.F.			L.P.			S.A.		B.C.		B.S.	
	$R_{1}$ (M$\Omega$)	$C_{1}$ ($\mu$F)	$C_{pc}$ ($\mu$F)	$R_{ct}$ (M$\Omega$)	$C_{pc}$ ($\mu$F)	$R_{ct}$ (k$\Omega$)	W (k$\Omega$)	$C_{pc}$ ($\mu$F)	$R_{ct}$ (k$\Omega$)	W (k$\Omega$)	$C_{pc}$ ($\mu$F)	$R_{ct}$ (k$\Omega$)	$C_{pc}$ ($\mu$F)	$R_{ct}$ (k$\Omega$)	$C_{pc}$ ($\mu$F)	$R_{ct}$ (k$\Omega$)
10	74.30	1.62	1.27	8.66	4.00	15.30	0.69	2.56	23.9	1.09	2.50	9.36	1.57	35.0	2.50	8.91
50	56.00	2.12	1.37	8.32	4.26	14.80	0.81	2.73	23.10	1.27	7.13	10.26	1.54	35.7	7.13	8.92
250	51.6	2.34	1.36	7.60	4.77	15.2	0.82	3.05	23.7	1.29	3.39	10.79	1.53	35.30	3.39	8.82
2900	41.10	2.64	1.41	6.37	5.02	15.00	0.77	3.21	23.4	1.22	6.48	11.38	2.10	31.2	6.48	7.01
5400	34.30	2.7	1.47	6.21	4.08	15.20	0.33	2.61	23.70	0.52	5.48	11.43	2.34	31.8	5.48	7.95
50400	20.30	2.92	1.56	4.76	3.98	15.20	0.33	2.57	23.90	0.53	5.57	10.89	2.36	25.8	5.57	6.57
200400	9.66	3.06	1.81	2.10	3.62	15.4	0.34	2.32	23.99	0.54	5.98	10.50	2.84	21.8	5.98	5.42
500400	6.66	3.88	2.09	1.68	3.56	14.00	0.34	2.28	21.82	0.37	5.35	11.27	2.92	21.8	5.35	5.44

From the Table 1, it is evident that in case of E. coli, $R_{1}$ has monotonic decrease which states that with increase in bacteria concentration, the conductance at the electrode interface increases. In Table 2 we have compared our results with the existing literature.

**Table 2:** Comparison table showing detection techniques, materials used, and their detection limits for various sensing platforms.

S. no.	Technique	Material	Detection limit	References
1	Fluorescence	Carbon Dots and Au NPs	$1.03 \pm 3.54$ nM	^42^
2	Fluorescence	Fluorescent carbon spindles (FCS)	$0.38 \times 10^{-7}$ M	^43^
3	ELISA	Paper-ELISA assay	$1 \times 10^4$ CFU/mL	^44^
4	ELISA	Magnetic nanoparticles (MNPs)	$2.6 \times 10^5$ CFU/mL	^45^
5	Potentiometry	*E. coli* CECT 675 aptamers with SWCNT	26 CFU/mL	^46^
6	EIS	Graphene modified Au electrodes with anti-fimbrial *E. coli* antibodies	10 CFU/mL	^35^
7	EIS	Graphene/aminoterephthalic acid electrode with anti-*E. coli* antibodies	2 CFU/mL	^36^
8	EIS	Au/Graphene modified with anti-*E. coli* O157:H7 antibodies	150 CFU/mL	^37^
9	EIS	Multilayer LB film of poly(lactic-co-glycolic acid) coated FeO nanoparticles modified with monoclonal antibodies specific to *Escherichia coli* O157:H7	3 CFU/mL	^47^
10	EIS	Single layer LB film of ODA-Gr with no bioligands	2.5 CFU/mL	This work

From the Table 2 we can observe that most of the available platforms or techniques use bio-labels such as antigen or antibodies for selective detection of E. coli. Meanwhile, our work showed higher or comparable detection limit without any modification on the graphene surface with bio-ligands. Due to the distinct impedance responses observed for different classes of bacteria, the data can be utilized to train machine learning algorithms, enabling the creation of a classification map for robust bacterial sensing. To evaluate this approach, various machine learning algorithms were tested for their effectiveness in classifying bacterial strains based on raw impedance data. The algorithms were implemented using the scikit-learn Python library. The raw impedance data was split into training and testing sets, with 80$\%$ of the data used to train the machine learning model and 20$\%$ reserved for evaluating its performance. Table 3 summarizes the machine learning algorithms tested for bacterial classification and compares their prediction accuracy.

**Table 3:** Different ML classifiers and their prediction parameters for the raw impedance data for six bacteria culture.

Classifier	Bacteria type	Precision	Recall	F1-score	Classification Accuracy
Random Forest	B.C.	0.44	0.47	0.45	71%
	S.A.	0.92	0.91	0.91	
	P.F.	0.59	0.54	0.57	
	**E. coli**	**1**	**1**	**1**	
	L.P.	0.84	0.78	0.81	
	B.S.	0.51	0.57	0.53	
Support Vector Machine	B.C.	0.06	0.01	0.01	20%
	S.A.	0	0	0	
	P.F.	0	0	0	
	**E. coli**	**0.86**	**0.29**	**0.43**	
	L.P.	0.15	0.08	0.1	
	B.S.	0.17	0.9	0.28	
K Nearest Neighbours	B.C.	0.19	0.37	0.25	35%
	S.A.	0.43	0.42	0.42	
	P.F.	0.24	0.23	0.24	
	**E. coli**	**0.95**	**0.62**	**0.75**	
	L.P.	0.41	0.21	0.27	
	B.S.	0.29	0.27	0.28	
Decision Tree	B.C.	0.55	0.56	0.55	77%
	S.A.	0.92	0.91	0.91	
	P.F.	0.68	0.65	0.66	
	**E. coli**	**1**	**1**	**1**	
	L.P.	0.85	0.81	0.83	
	B.S.	0.64	0.69	0.66	
Gradient Boost Accuracy	B.C.	0.28	0.26	0.27	67%
	S.A.	0.43	0.13	0.2	
	P.F.	0.31	0.28	0.29	
	**E. coli**	**1**	**0.49**	**0.66**	
	L.P.	0.23	0.42	0.29	
	B.S.	0.3	0.46	0.36	
Bagging Classifier	B.C.	0.69	0.7	0.69	83%
	S.A.	0.94	0.95	0.94	
	P.F.	0.73	0.73	0.73	
	**E. coli**	**1**	**1**	**1**	
	L.P.	0.93	0.94	0.93	
	B.S.	0.71	0.72	0.72	
Gaussian Naive Bayes	B.C.	0.52	0.18	0.27	26%
	S.A.	0.19	0.68	0.3	
	P.F.	0.08	0.01	0.02	
	**E. coli**	**0.93**	**0.25**	**0.4**	
	L.P.	0.27	0.34	0.3	
	B.S.	0.21	0.06	0.1	
Multilayer Perceptron Neural Network	B.C.	0.42	0.26	0.32	34%
	S.A.	0.27	0.58	0.37	
	P.F.	0.5	0.09	0.15	
	**E. coli**	**0.91**	**0.37**	**0.53**	
	L.P.	0.36	0.32	0.34	
	B.S.	0.25	0.46	0.32	

**Fig. 3:**
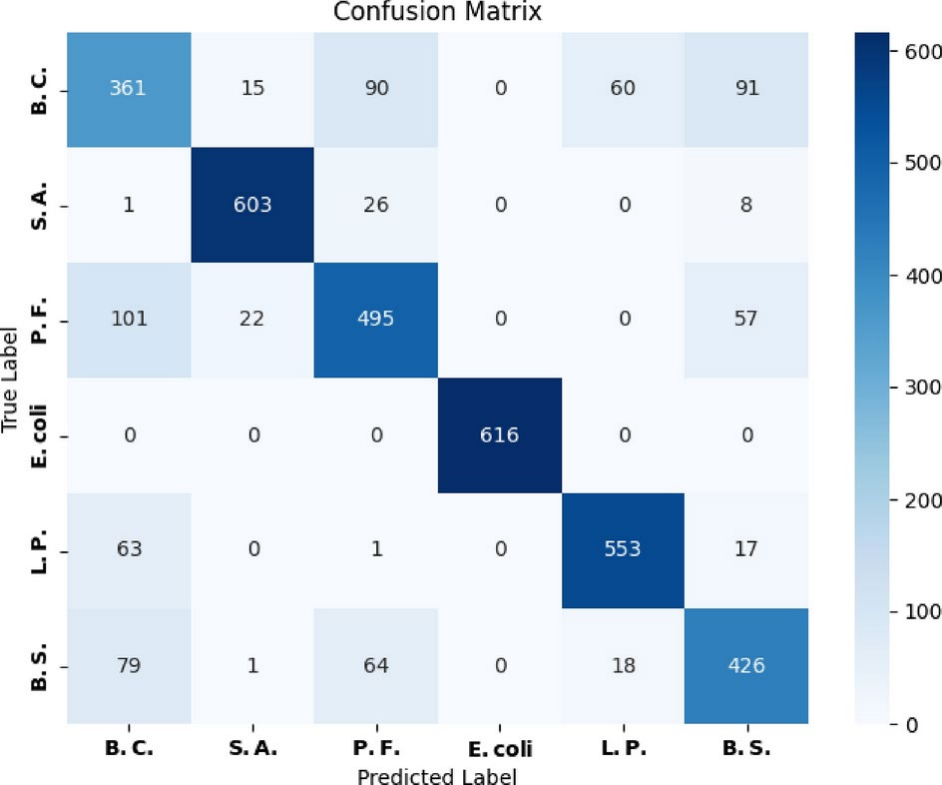
Confusion matrix for Bagging classifier.

Bagging classifier and Decision tree were comparatively more suitable algorithm for the classification for our data with $83~\%$ and $77~\%$ accuracy respectively. It is also evident that most of the ML classifiers predicted E. coli better compared to other five bacteria. The multiclass classification for the 6 bacteria for the best classifier is shown in the confusion matrix in Fig 3. The confusion matrix also showed zero false positive values for E. coli.

Figure 3 presents the confusion matrix for the best-performing classification algorithm for our data, the Bagging Classifier. From the confusion matrix, it is evident that E. coli achieved perfect classification, with all true positive values and zero false positives; specifically, 616 instances of E. coli were correctly classified without any misclassifications. The second-best classification performance was observed for the S.A., where 603 instances were correctly classified. However, 26 instances were misclassified as P.F. (Gram-negative) and additional 8 and 1 instances were misclassified as B.S. and B.C., respectively. The poorest classification was observed for B.C., which had the fewest true positives and false positives distributed across all other bacteria, except for E. coli. Thus, as demonstrated in Fig. 3, machine learning enables accurate and selective detection of E. coli based on raw impedance responses. The application of a well-trained machine learning algorithm significantly reduces analysis time and eliminates the possibility of human error, provided the model is rigorously trained with high-quality data.

## Conclusion

This study demonstrates the potential of impedance-based sensing combined with machine learning for the selective detection and classification of bacterial strains. The distinct impedance profiles arising from the unique interactions of Gram-positive and Gram-negative bacteria using ultrathin films of ODA-functionalized graphene provide a robust foundation for bacterial discrimination. The findings reveal that Gram-positive bacteria, due to their clustering and biofilm formation, exhibit nonlinear impedance responses, while Gram-negative bacteria show additional complexities in their impedance profiles, necessitating distinct circuit elements for modeling. The pronounced differences in the EIS spectra of E. coli relative to other bacteria provide a robust basis for its selective detection, emphasizing the specificity and reliability of the developed sensing platform. The results highlight the exceptional selectivity of this approach for E. coli, achieving perfect classification with no false positives using the Bagging Classifier. Given the critical role of E. coli as an indicator organism for water and food safety and its association with serious infections, the ability to selectively and accurately detect E. coli is of paramount importance. The integration of machine learning algorithms not only enhances classification accuracy but also significantly reduces analysis time, eliminating human errors through automated processing. This work establishes a robust framework for the rapid and selective detection of E. coli and other bacterial strains based on raw impedance responses. The findings pave the way for developing cost-effective, scalable diagnostic tools, with future efforts directed toward expanding datasets, optimizing machine learning pipelines, and applying this methodology to complex biological and environmental samples.

## Methods

**Synthesis of ODA-Gr**: Reduced graphene oxide (rGO) was synthesized using modified Hummer’s method. 1g of graphite powder and 0.5 g of $NaNO_{3}$ were taken in a beaker. To this mixture, 23 mL of $H_{2}SO_{4}$ was added slowly. The beaker was kept in the ice bath to maintain the temperature of the solution at $0^{\circ }$C under constant stirring. The solution was stirred for about an hour to get homogeneous solution. Further, 3g of $KMnO_4$ was added slowly so that temperature of the solution did not exceed $20~^{\circ }$C while constantly stirring in an ice bath. Then, the solution was stirred thoroughly while maintaining the temperature of the solution at $35~^{\circ }$C. After 12 hrs, 500 mL of distilled water was added slowly to the above solution to stop the oxidation process. Simultaneously, 5mL of 30$\%$ $H_{2}O_{2}$ was also added and stirring was continued for 5-10 min to terminate the reaction. The solution was cooled to room temperature and washed with 10$\%$ HCl and distilled water alternatively for 3 times. The precipitate was filtered and dried at $60^{\circ }$C in air. The final product was obtained as reduced graphene oxide (rGO). Further, 100 mg of rGO was dispersed in 100 mL of ethanol containing 100 mg of ODA. The suspension was sonicated for 2 h and then stirred for 24 hrs at room temperature. The precipitate was filtered and washed with ethanol to remove excess ODA. The precipitate was centrifuged and washed with distilled water and ethanol multiple times to remove the excess ODA. The precipitate was finally dried at $60~^{\circ }C$ in air. The final product was named as octadecylamine reduced graphene oxide (ODA-Gr).

### LB film preparation

The LB films were prepared in Langmuir-Blodgett trough. Firstly, the trough was cleaned with de-ionized (DI) water (18.2 M$\Omega -cm$) DI water and chloroform successively. After cleaning, the LB trough was filled with DI water. Further, the gold substrate was cleaned thoroughly by ultrasonicating it in ethanol solution for 30 mins. After thorough cleaning, the substrate was attached to the dipper of the LB trough. The gold substrate was kept immersed inside the DI water prior to the monolayer formation at the air-water interface. A stock solution of 0.05 mg/mL ODA-Gr was prepared in absolute ethanol. Thereafter, 500 $\mu$L from the stock solution was spread onto the water surface between the two barriers of the Langmuir trough (KSV-NIMA). The system was left undisturbed for 30 minutes to allow the solvent to evaporate and to get a homogeneous spreading of ODA-Gr. Further, the Langmuir film of ODA-Gr was compressed by symmetric lateral movement of the barriers, and the corresponding surface pressure $\pi$ as a function of area occupied by the film was recorded. The surface pressure was recorded using a pressure sensor (accuracy = 0.01 mN/m) which was connected with a Wilhelmy plate (filter paper). The surface phases of the Langmuir film were observed using a Brewster angle Microscope (BAM,KSV NIMA Micro-BAM). The microscope was equipped with 30 mW laser with a wavelength of 659 nm. The spatial resolution of the BAM images is 12$\mu$m. The Langmuir film was then transferred on to gold electrodes via LB method at the target surface pressure ($\pi _{t}$) of 15mN/m. The dipper speed was maintained at 1 mm/min. The target surface pressure was kept constant via controlled feedback mechanism of barriers for efficient film deposition.

### Bacteria culture

The common and representative bacterial strains of gram +ve (Staphylococcus aureus subsp. aureus MTCC1430T (S.A.), Bacillus cereus MTCC430 (B.C.), Bacillus subtilis MTCC121 (B.S.)) and gram -ve groups (Escherichia coli DH5 alpha MTCC1652 (E. coli), Pseudomonas fluorescens MTCC103T (P.F.), Legionella pneumophila subsp. pneumophila ATCC 33152-0211P (L.P.)) were procured from the culture collection at MTCC, IMTECH, Chandigarh, India (https://mtccindia.res.in) and Biomall(https://www.biomall.in). The bacterial cultures were sub-cultured in suitable medium as per supplier’s instructions. The viable cell count of each culture was determined using CFU count methods and concentration of the culture was expressed as CFU/ml. In addition, the freshly grown bacteria culture was serially diluted and 100 $\mu$L of this culture was spread on solid media. After overnight incubation, plates that had a countable range of colonies were used for colony counting. The CFU number was also counted for each strain as per Sieuwerts et al.^48^. Serial dilution was performed on each bacteria culture to obtain a desired concentration for sensing measurements.

### Sensing measurement

Electrochemical impedance spectroscopy (EIS) was utilized for sensing measurements in this study. LB deposited ODA-Gr/Gold electrodes served as the working electrode, while an Ag/AgCl electrode was employed as the reference electrode, and a platinum wire as the counter electrode. The standard phosphate buffer saline (PBS) electrolyte was utilized for sensing measurement. Each bacterial culture was added sequentially to the electrolyte, and impedance measurements were conducted across a frequency range of 10 mHz to 1 MHz for each sample. Impedance measurements of each bacteria were taken every hour for 12 hours at the highest concentration. Such plot for E. coli bacteria is added in the Supplementary Information. We also verified the reproducibility by repeating the experiments with newly fabricated films and got a ±4$\%$ variation in the results.

## Supplementary Information

Supplementary Information.

## Data Availability

All data generated or analysed during this study are included in this article. These data can also be made available from the corresponding author upon reasonable request.
