# Supplementary material for: Synergistic detection of E. coli using ultrathin film of functionalized graphene with impedance spectroscopy and machine learning
Source: Sci Rep. 2025 Apr 30;15:15177. doi: 10.1038/s41598-025-00121-3 (PMC12043823; doi:10.1038/s41598-025-00121-3)
Supplement: Supplementary file 1 — Supplementary Information. [file 41598_2025_121_MOESM1_ESM.pdf]

Synergistic Detection of E. Coli Using Ultrathin Film of Functionalized Graphene with Impedance Spectroscopy and Machine Learning

Amrit Kumar, Shweta Mishra, R. K. Gupta and V. Manjuladevi*
Department of Physics, Birla Institute of Technology and Science, Pilani (BITS Pilani), 333031 Rajasthan, India
**raj@pilani.bits-pilani.ac.in*

<u>Supplementary Information (S1)</u>

Characterization of synthesized ODA-Gr:

The powder Xray diffraction (XRD) pattern of as synthesized rGO and ODA-Gr samples is as shown in Fig S1. A peak observed at $2\theta = 26.4\,°$ corresponding to (002) plane, indicates the formation of reduced GO by restoring the pi-conjugated structure of graphene. The poor arrangement could be caused by the formation of a nearly single layer of rGO after being reduced from GO. The d-spacing is found to be 0.34 nm which shows that oxygen-containing functional groups are removed efficiently. The intense peak at $2\theta = 21.3\,°$ shows increased d-spacing of 0.42 nm due to functionalization increasing the layer spacing in the presence of ODA functional groups and hybrid structure formation. The existence of strong van der Waals' forces between each layer makes the final product to be a few layers thick ODA-Gr instead of a formation of a single layer. A small, less intense peak is observed at the $2\theta = 42.9\,°$ corresponding to (001) plane orientation and is attributed to the turbostratic band of disordered carbon materials. Retention of graphitic peaks highlights partial graphitic character, ensuring structural stability and enhanced material versatility.

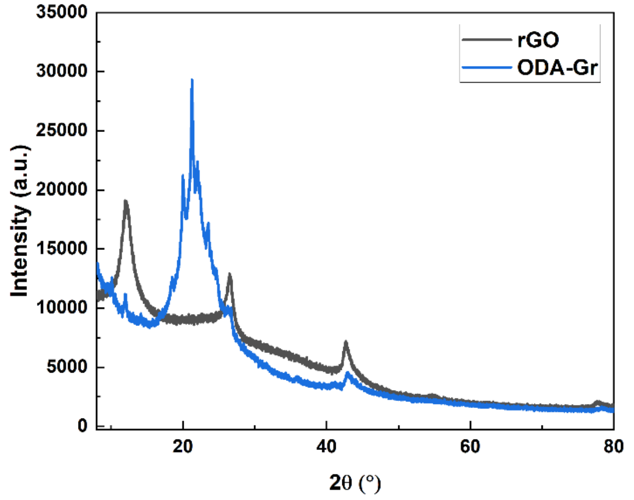

Figure S1. XRD pattern for rGO and ODA-Gr

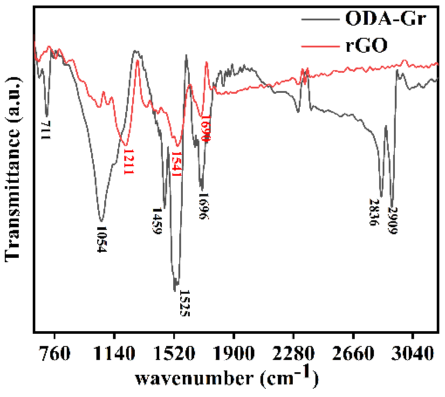

Figure S2. FTIR spectra for rGO and ODA-Gr

Fourier transform infrared (FTIR) spectra of rGO and ODA-Gr is presented in Fig S2. The broad peak of rGO around 1690 cm^{-1} corresponds to C-O carboxyl stretching vibration and C-C in the aromatic ring. The peak around 1583 cm^{-1} typically for skeletal vibration of C=C domain is attributed to the crystallinity. The FTIR spectrum of ODA-Gr shows narrow absorption bands around 2836 and 2909 cm^{-1} together with the peak at 711 cm^{-1} corresponding to the symmetric and antisymmetric stretching of long aliphatic chain of ODA. Meanwhile, the FTIR spectrum of rGO does not show such peaks. Hence, the functionalization of ODA is confirmed on the rGO surface.

The field emission scanning electron microscope (FESEM) images of as synthesized rGO and ODA-Gr are shown in Fig S3. The formation of graphene sheets is clearly visible. The FESEM images (a) show the presence of wrinkles and scrolls in the rGO structure, suggesting the formation of few-layered graphene sheets. This characteristic is because of the flexible nature of the rGO. The transparency of sheets indicates formation of thin rGO sheets. There is negligible difference in the FESEM images of rGO and ODA-Gr (b) showing no morphological differences arising after the functionalization of rGO with ODA.

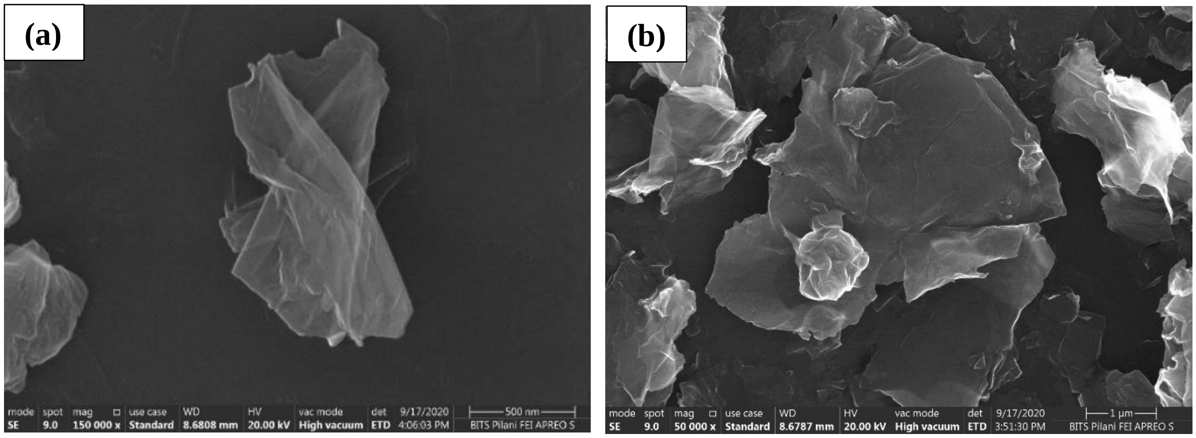

Figure S3. FESEM image of as synthesized (a) rGO (b) ODA-Gr

Characterization of LB film of ODA-Gr:

Xray reflectivity (XRR) measurements were carried out to calculate the thickness of the LB film of ODA-gr transferred on solid substrate. The XRR curve along with the simulated curve is shown in Fig. S4.

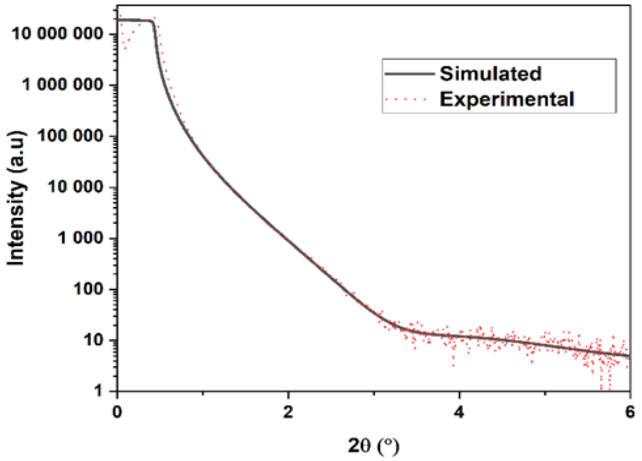

Figure S4. X-ray reflectivity image for the observed and fitted data.

The thickness of the LB film was calculated by fitting the data using SmartLab Studio software from Rigaku. Here we have used Parratt's formalism for fitting. We repeated the XRR measurements at different places to account for the homogeneity of the monolayer on the electrode. The simulated spectra showed the average thickness of 2.47 nm ± 0.05 nm.

Experimental Flowchart:

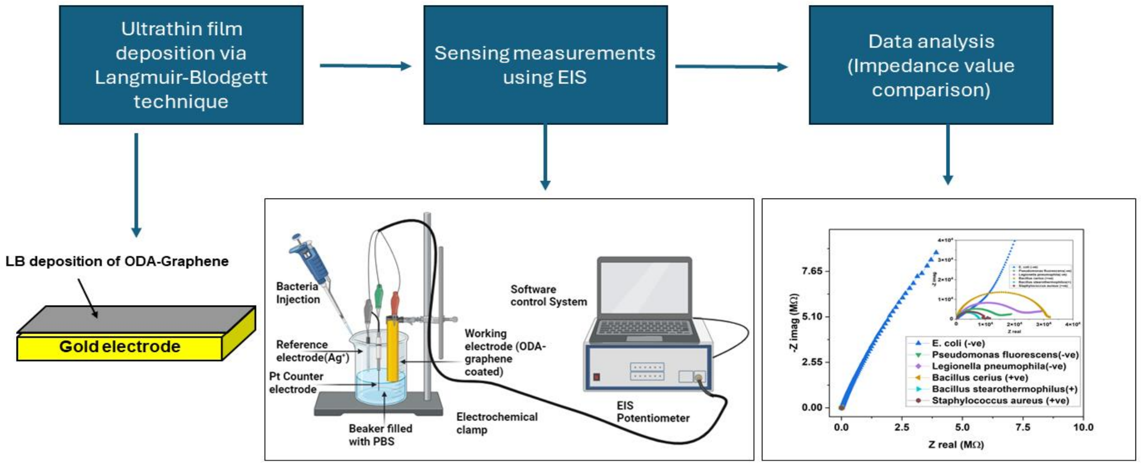

Figure S5. Flowchart of the study.

Repeatability: Impedance spectra for different intervals up to 12 hours for E. coli are shown in Fig S6.

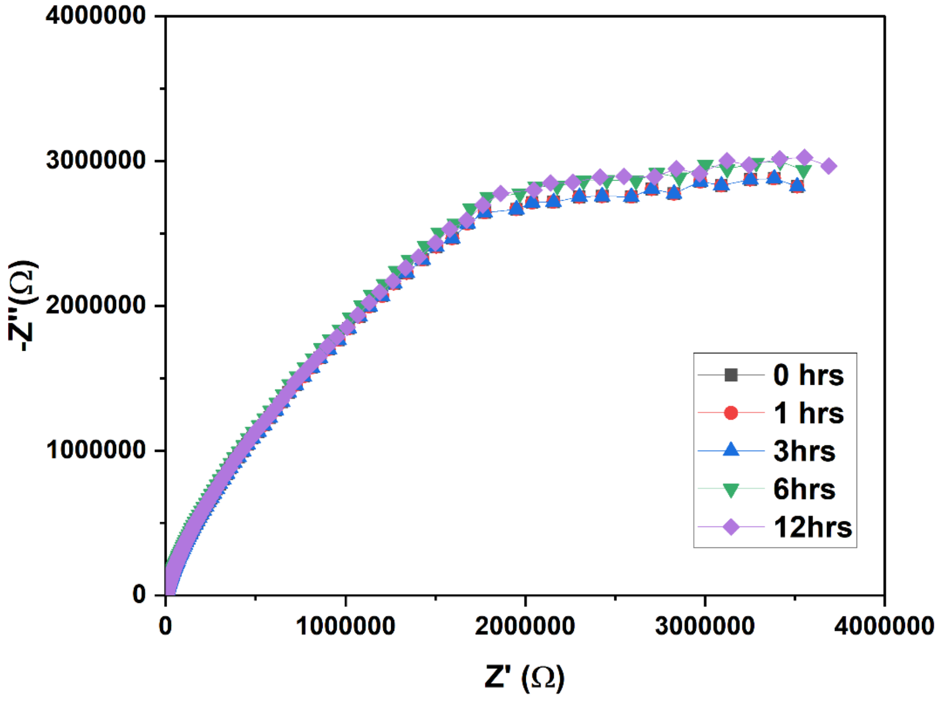

Figure S6. Nyquist plot for different time intervals for E coli bacteria.

The impedance measurement for an E. Coli bacterial concentration of 500400 cfu/mL at various time intervals is displayed in the Nyquist plot in Fig. S6. Up to three hours, the impedance measurement revealed minimal fluctuation. Then, as the duration was extended, a slight variance was noticed. It was found that the overall impedance variation ranged from ~ 3 to 7 percent.
